# Early identification of substandard breast screening performers

**DOI:** 10.1186/bcr2965

**Published:** 2011-11-04

**Authors:** L Dong, Y Chen, AG Gale

**Affiliations:** 1 Loughborough University, Loughborough, UK

## Introduction

All UK breast screeners can voluntarily undertake the PERFORMS scheme, where they examine recent screening cases receiving immediate feedback. Once all individuals have participated, data are calculated on how they performed compared with peers allowing poor performers to be identified. A way of potentially identifying such poor performers much earlier is proposed.

## Methods

Information from the last round of the PERFORMS scheme was reanalysed for which the low performance threshold value was known. Data for randomly selected small groups of participants were repeatedly bootstrapped with the aim of artificially determining a threshold of low performance and comparing this with the known actual threshold. Using a varying number from four to 50 participants, a sample of 1,000 randomly selected small groups was constructed for each number of participants. After bootstrapping each small group, a distribution of 1,000 thresholds of low performance was constructed and median values and standard errors of this distribution calculated to determine how the number of participants affected the estimation accuracy.

## Results

The standard error of the estimated threshold reduced as group size increased, indicating better estimation accuracy. Using data from as few as 10 people the artificial threshold approached the known actual threshold of poor performance.

## Conclusion

Individuals who are performing less than their peers on the scheme can be identified early without all screeners having first taken part. Whilst not an absolute outlier measure, this information can be fed back in a timely manner, so enabling the individual to improve their cancer identification performance.

